# Cunean Tenectomy

**Published:** 1898-08

**Authors:** 


Cunean Tenectomy is particularly effective in prominent spavin, and after repeated counter-irritation has failed to relieve the lameness. Even as a first resort, statistics gathered at the clinics show a greater percentage of recoveries than all other forms of treatment combined.

